# IMNGS: A comprehensive open resource of processed 16S rRNA microbial profiles for ecology and diversity studies

**DOI:** 10.1038/srep33721

**Published:** 2016-09-23

**Authors:** Ilias Lagkouvardos, Divya Joseph, Martin Kapfhammer, Sabahattin Giritli, Matthias Horn, Dirk Haller, Thomas Clavel

**Affiliations:** 1 ZIEL Institute for Food and Health, Core Facility NGS/Microbiome, Technische Universität München, Freising, Germany; 2 Department of Microbiology and Ecosystem Science, University of Vienna, Vienna, Austria; 3 Chair of Nutrition and Immunology, Technische Universität München, Freising, Germany

## Abstract

The SRA (Sequence Read Archive) serves as primary depository for massive amounts of Next Generation Sequencing data, and currently host over 100,000 16S rRNA gene amplicon-based microbial profiles from various host habitats and environments. This number is increasing rapidly and there is a dire need for approaches to utilize this pool of knowledge. Here we created IMNGS (Integrated Microbial Next Generation Sequencing), an innovative platform that uniformly and systematically screens for and processes all prokaryotic 16S rRNA gene amplicon datasets available in SRA and uses them to build sample-specific sequence databases and OTU-based profiles. Via a web interface, this integrative sequence resource can easily be queried by users. We show examples of how the approach allows testing the ecological importance of specific microorganisms in different hosts or ecosystems, and performing targeted diversity studies for selected taxonomic groups. The platform also offers a complete workflow for *de novo* analysis of users’ own raw 16S rRNA gene amplicon datasets for the sake of comparison with existing data. IMNGS can be accessed at www.imngs.org.

Microbial communities are key members of many ecosystems on earth. They play important roles in essential biological functions ranging from carbon and nitrogen cycles in the environment to regulation of immune and metabolic responses in colonized animal and human hosts^1^,^2^. Hence, research in microbial ecology is essential for proper understanding of these communities and their functional impact.

One critical aspect in microbial ecology is to describe the members of microbial communities that, in contrast to macro ecology, cannot be easily identified and enumerated. Studies in the field had been limited in the past to culture-based approaches that allow isolation of microorganisms as pure cultures and downstream characterization *in vitro* and *in vivo*. The limitation of these approaches is that a substantial proportion of microorganisms escape cultivation^3^. Thus, early advances in sequencing technologies enabled culture-independent profiling of microbial communities using phylogenetic markers such as the highly conserved 16S ribosomal RNA (rRNA) gene^4^. Next Generation Sequencing (NGS) methods, including pyrosequencing and Illumina technology, have further revolutionized the field of microbial ecology by allowing in-depth and high-throughput parallel analysis of PCR products from multiple samples. These amplicon sequencing approaches have been used extensively for characterization of diverse ecosystems ranging from aquatic and terrestrial to host-derived ecosystems^5^,^6^,^7^.

The maturation of NGS technologies has allowed expansion of the number of parallel samples under investigation and enabled investigation of temporal dynamics in composition of microbiomes. Due to continuously increasing capacity and decreasing costs, recent sequencing-based studies were in the range of a few hundreds or thousands of samples per study^8^,^9^,^10^,^11^. These advances resulted in a flood of primary amplicon sequence data that are usually archived in one of the long-term repositories of the International Nucleotide Sequence Database Collaboration, including the Sequence Read Archive (SRA), for reference and reuse. These repositories have accumulated unprecedented amounts of 16S rRNA gene amplicon datasets that cover the vast majority of biomes on earth biospheres^12^. However, there is to date a lack of user-friendly and flexible approaches dedicated to standardized and reproducible meta-analysis of microbial distribution and diversity exploiting this large pool of sequence data. A web tool that can be used to analyze amplicon sequences in public archives has recently been published^13^, but the system relies on a relatively limited number of data and is not really extensible.

We previously demonstrated the potential of using accumulated amplicon data for estimation of the diversity of the bacterial phylum *Chlamydiae*^14^. In the present work, we describe the Integrated Microbial Next Generation Sequencing (IMNGS) web platform, a transparent and fully flexible system for integration of archived amplicon 16S rRNA gene sequence reads into readily accessible sample-specific databases. Scripts in the backend of IMNGS routinely check and retrieve raw data from SRA and uniformly process and classify them. Registered users can obtain sequences across all samples or lists of samples selected by the user, and investigate prevalence patterns based on similarity to selected sequences or taxonomic groups of interest. In addition, a user friendly implementation of the UPARSE amplicon analysis pipeline^15^ is offered for researchers that want to explore their own raw data and compare them to the similarly preprocessed samples in the IMNGS system. The present manuscript contains a detailed description of IMNGS and provides example analyses to demonstrate the potential of this novel platform.

## Results

### Features of the IMNGS platform

IMNGS is based on an innovative bioinformatic workflow embedded in a web interface, which allows users to perform large-scale analysis of all 16S rRNA gene amplicon datasets available in SRA, for instance to generate novel insight into the diversity and ecological distribution of specific bacterial groups of interest. IMNGS has two core functions (query types) that help generating such data: sequence similarity search against template sequences provided by the users, and query based on taxonomic classification of sequences available in the IMNGS database. A template analysis for each approach is presented in the next sections.

The first complete IMNGS crawling of the SRA detected 105,374 runs (samples) that matched the amplicon study description. After quality filtering and processing, 55,073 runs remained that became available for query (build 1510, i.e. October 2015, used in the present paper for description and demonstration purposes; the current size of the IMNGS database as per July 2016 is 85121 runs). To ensure a seamless integration of the continuous flow of new samples in SRA, the sequences from each sample are clustered *de novo* at 97% sequence identity. All samples represent currently a total of 1.4 billion sequences that clustered into 53,696,442 sample-specific OTUs (operational taxonomic units). This means that an OTU name (or number) has no reference value outside of the given sample where it was created. Although independent *de novo* clustering impairs more straightforward comparison between samples as could be done with a common OTU table, it has the advantage to allow continuous update of the database and to retain all unknown sequences that would be otherwise lost during closed reference-based clustering required for creation of such a common OTU table.

The origin of samples in IMNGS is diverse (96 categories), but not uniformly distributed. The database is skewed towards host-associated sources (67%), and particularly human samples (50%). Samples of environmental origin account for approximately 33% of the database. The performance of queries varies greatly depending on the number of samples selected. In general, a taxonomy query is finished within one hour, while a similarity query over all samples can take approximately 6 h per sequence. The analysis of newly generated 16S rRNA gene amplicon reads from users is fast, *i.e.*, returns OTU tables and corresponding sequence information from raw data in less than an hour. Readers are invited to look in detail at the methods for information on the scalability of the system.

As described in detail in the Methods section and in README files provided online after analysis, IMNGS delivers detailed information about the abundance and distribution of sample-specific OTUs that match queried sequences or taxonomic groups in the form of tables and sequence files. Further processing of data to generate figures (as presented below in the template analyses) is intentionally left in the hands of users, because it requires downstream analyses that are case-dependent and must be adjusted according to the respective study purposes. The next results sections present two template analyses meant to exemplify the types of research questions addressable by using IMNGS-derived data.

### Host specificity of the bacterial species *Acetatifactor muris*

**Figure 1:**
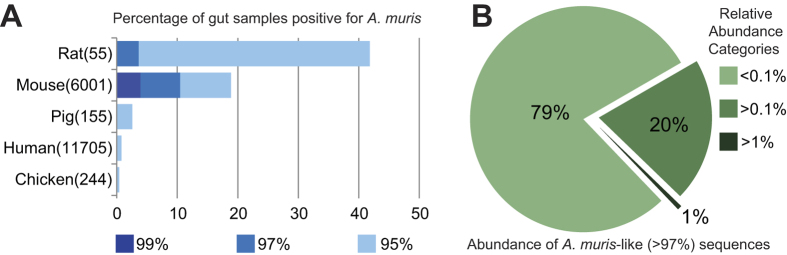
Host range and relative sequence abundance of bacteria related to *Acetatifactor muris* based on sequence similarity search in IMNGS. (**A**) Prevalence of *A. muris*-like sequences at different levels of similarity expressed as percentages of positive samples in each host (the number of samples included in the analysis are shown in parenthesis). (**B**) Percentages of samples positive for the species *A. muris* (97% sequence similarity) at the indicated threshold of relative abundances.

The gut microbiota has been implicated in the regulation of host metabolic responses in obesity and chronic metabolic disorders^16^,^17^. Mouse models of obesity are helpful to study molecular mechanisms underlying microbe-host interactions, but very few key bacterial species regulating host metabolic responses have been identified^18^,^19^,^20^. The type strain of *Acetatifactor muris* was originally isolated from the intestine of an obese mouse and represents so far the sole described species of the genus^21^. Although we have repeatedly detected changes in the occurrence of molecular species phylogenetically related to *A. muris* in diet-induced mouse models of obesity as measured by high-throughput sequencing^22^ (and unpublished data), there is to date no information about their overall host specificity. Therefore, we used a similarity query in IMNGS to determine the ecological characteristics of this novel group of bacteria. The output of this large-scale amplicon search (including 58,522 sequences originating from 1,253 samples and a cumulative number of 2,332 OTUs) showed that bacteria with over 95% identity to *Acetatifactor* (approximately the genus level similarity cutoff) have a strong association with gut environments in rodents (Fig. 1A). This association became more specific to the mouse gut for sequences with more than 97% identity (commonly used as a conservative within-species identity cut off for full 16S rRNA gene sequences) and was even unique for mice for highly similar amplicon sequences (more than 99% identity). At the 97% (“species”) level, the observed relative abundance of *A. muris*-like sequences in positive samples was low, in general below 1% total sequence reads, suggesting that this bacterium appear at low abundance in most gut environments (Fig. 1B). However, the highest relative abundance of *Acetatifactor*-like 16S rRNA gene amplicons using 95% blast similarity as a cutoff were detected in sample SRR1210011 originating from a high-fat diet feeding study in mice^23^, reaching >8% total sequences. The next highest abundances (ca. 4% total reads) originated also from a mouse model of diet-induced obesity^24^. Tracing back those hits revealed OTUs with pairwise similarity ranging from 95 to 97%, indicating possible novel species of *Acetatifactor*. Interestingly, when corresponding pretreatment samples (before high-fat diet feeding) were examined, *Acetatifactor-*like amplicons were barely or not detected, supporting an association between the presence of *Acetatifactor* spp. and diet-induced obesity in mice.

### Diversity of the phylum *Poribactreria*

**Figure 2:**
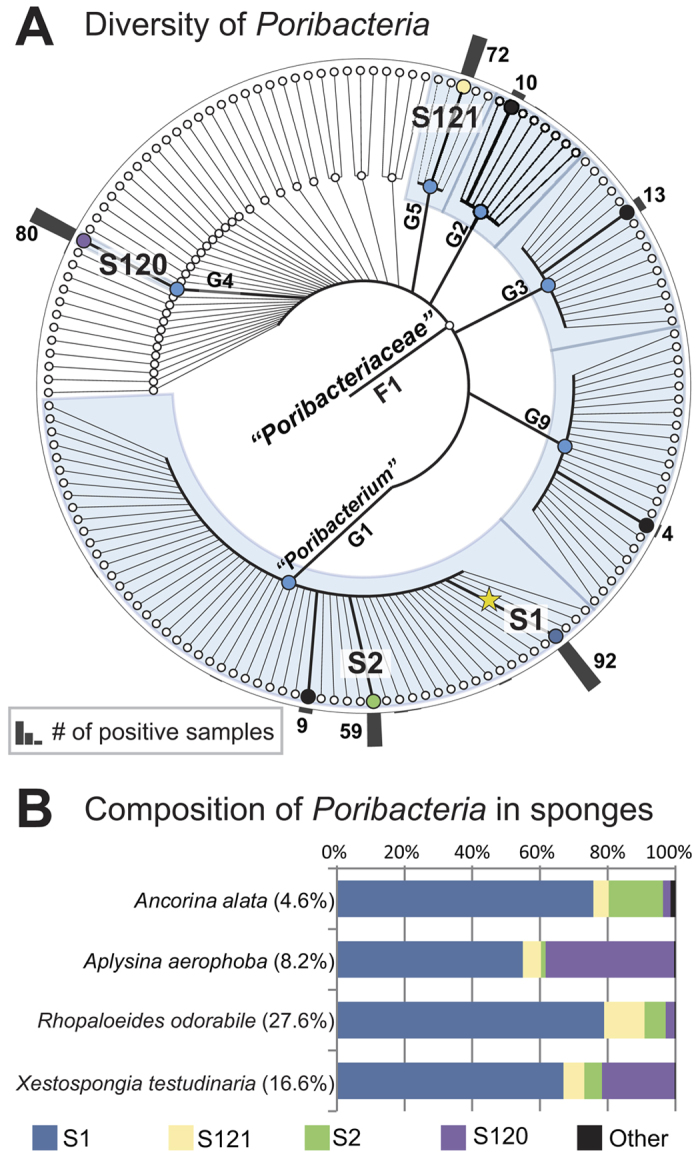
Population structure and diversity of the bacterial phylum *Poribacteria* based on a taxonomic query in IMNGS. (**A**) Successive clustering of *Poribacteria* sequences (n = 2,308) at different levels of sequence similarity (~3, 5 and 10%). Prominent molecular species and genera clusters based on the number of samples they represented in the database (black bars) are written in bold and indicated by colored nodes. The star represents the candidate type species of *Poribacteria* originally investigated by single-cell genomics^28^. (**B**) Host specificity of sponge colonization by *Poribacteria*. Average contribution of each prominent molecular species of *Poribacteria* relative to the total number of sequences classified as *Poribacteria* in the different sponge species. The maximum relative sequence abundance of *Poribacteria* in the microbial profiles of each sponge is shown in parenthesis (the rest corresponded to other bacteria).

*Poribacteria* is a bacterial phylum that was first identified as an important constituent of microbial communities associated with marine sponges^25^. *Poribacteria* serve as model organisms to understand the interaction between sponges and their diverse microbial community, and they share a number of unique features such as a compartmentalized cell and a high number of eukaryotic-like proteins^26^. To date, no cultivable strain exists but extensive genetic information has been obtained by single cell genomics^27^,^28^. Beside sponges or water and sediment samples in the vicinity of sponges, reports on the occurrence of *Poribacteria* have been scarce, pointing at a rather strict and intricate association between these bacteria and their host^29^,^30^. Here, we exploited the accumulated sequence data in IMNGS to investigate global diversity and population structure of *Poribacteria*. The taxonomic query resulted in 86,293 sequences originating from 200 samples and a cumulative number of 2,425 OTUs classified as *Poribacteria*. To maximize the sequence space for analysis, we also added all GenBank-derived 16S rRNA clones classified as *Poribacteria*. The complete set of sequences was aligned and trimmed around the highest coverage region resulting in a total of 2,308 sequences with >370 nucleotides. These sequences were clustered at different similarity levels resulting in a comprehensive view of *Poribacteria* sequence diversity (Fig. 2A). The most conservative clustering produced by CROP supported the existence of 151 molecular species clusters at ~3% similarity (S1-S151). These clusters further assembled in 38 molecular genera at ~5% similarity (G1-G38), and finally in one single molecular family at ~10% similarity (F1). The diversity of *Poribacteria* can be summarized in 8 major (most dominant) molecular species clusters distributed in 6 genera. Looking at the contribution of each of these 8 species in samples that were most abundant in *Poribacteria* sequences, we observed no strict host specificity for a particular sponge species (Fig. 2B). Cluster S1 was detected across 92 samples and was the most prevalent in all sponge species, while sequences belonging to cluster S120 showed some preference for the host species *Aplysina aerophoba* and *Xestospongia testudinaria*, with 38% and 22% average abundance, respectively.

## Discussion

IMNGS is currently the biggest and most detailed open resource of processed 16S rRNA gene amplicon datasets available to date. The user friendly interface guarantees easy and rapid access to the resource. The provided output files return detailed information on the distribution of specific taxa or OTUs across a broad range of diverse samples available through IMNGS. Results will be of use to researchers interested in testing the occurrence and diversity of any specific bacteria of interest in the broad fields of environmental research, clinical microbiology, and microbial ecology. In the present manuscript, we demonstrated major features of IMNGS by conducting two showcase studies on the occurrence of the bacterial genus *Acetatifactor* in diet-induced obesity and on global diversity of the important sponge symbionts *Poribacteria*.

In contrast to diversity analysis where the source of sequence is partly irrelevant, interpretation of sequence distribution patterns depends heavily on the subset of samples selected for each analysis. Samples that are not representative of normal conditions in a given environment but rather correspond to extreme perturbations or exceptional cases may distort the ecological view of prevalence and abundance (e.g. gut profiles after antibiotic usage not specified in the sample description from SRA). Hence, one current limitation of the resource is related to the poor quality of metadata information about samples in SRA, which complicates interpretation of results if some of the samples under investigation are not representative of native ecosystems. That is why it is worth investing time in building a curated list of homogenous samples that better represent the question at hand. The list exporting and sharing functionality of IMNGS (see methods) helps the production and maintenance of highly curated lists of samples for various environment categories. On the other hand, studies oriented in exploring the global diversity of a particular taxonomic group should benefit from maximal retrieval of targeted sequences using all available samples.

Given a curated list of samples, similarity-based searches offer valuable insight in the distribution of query-like sequences within and across samples. In the example of *A. muris*, the observed patterns of prevalence supported its origin as a mouse gut-specific bacterial species^21^. At the genus level, *Acetatifactor* spp. showed also a strong host specificity of colonization in rodents and indicated clear abundance levels associated with high-fat diet feeding in mice. These hypotheses generated via IMNGS searches can now be tested experimentally by studying the molecular basis of this tight host adaptation and the role of *Acetatifactor* species in obesity models. Hence, IMNGS can now be used as a routine approach to extract information on the ecology of newly described microbes or known species for which such information is lacking.

Taxonomy-based queries with IMNGS are generally faster than sequence similarity-queries and can be useful for extraction of both ecological patterns and the available sequence space of selected taxa from phyla to families. The way of analysis of these data is open to the investigator depending on scientific questions to be addressed. Here, we provided an example of a diversity analysis for the phylum *Poribacteria* showing evidence for a sponge-associated bacterial clade with low host specificity. Different sponges can host a mixture of diverse *Poribacteria* sequences, although colonization is dominated by 4 main molecular species. The presence of only one main family level group within the phylum that contains also a low number of genera and species indicate an overall low diversity within the phylum. This is surprising considering the evolutionary time since divergence of *Poribacteria* from the common bacterial ancestor, which happened before diversification of the *Planctomycetes, Verrucomicrobia, Chlamydiae* superphylum^26^. Perhaps the restricted and evolutionary stable niche as well as vertical mode of transmission of these microorganisms did not favor diversification^31^. Of course, one should keep in mind that technology and primer sets can have a substantial impact on results^32^ and we cannot exclude that other families of *Poribacteria* that were not captured by the used primers or sequencing protocols exist.

Another web tool that exploits amplicon sequences in public archives has been published^13^. However, the IMNGS platform is a transparent and flexible integration of all 16S rRNA gene amplicon studies available in SRA. With currently >85,000 samples processed, representing approximately 2 billion sequences (build 1607), IMNGS is to the best of our knowledge the biggest and most detailed open resource of processed bacterial profiles, which is updated automatically. The existing tool mentioned above is limited to pyrosequencing studies representing a little more than 1.5 million sequences, and the database is not updated regularly. Moreover, it relies on BLAST similarity queries only, so predictions for taxonomic levels below the genus level can be imprecise. The clear and intuitive web front of IMNGS enables rapid execution of both similarity and taxonomic queries in a secured and personalized manner. The simplicity and power of the method will facilitate its implementation for a broad range of applications. For instance, rapid retrieval of ecological information on every new isolate or known species of particular clinical or environmental relevance is possible. Furthermore, the capacity for detailed analysis of universal bacterial diversity for any selected taxon of interest has no precedent in the field. One major feature of IMNGS is the list-based system where users can select specific samples of particular interest, which contrast to the published system where all sequences were merged together, which decreased search time requirements but comes with the major drawback that sample-specific information is lost. Finally, the user friendly implementation of UPARSE in IMNGS enables direct comparison of users’ own samples with existing datasets. As an independent solution for processing raw sequencing data, this analysis function in IMNGS has the advantage to be user friendly, i.e. it has a clear interface and no installation requirements. However, it can be restrictive for expert users and dedicated platforms such as QIIME^33^ and MOTHUR^34^ offer greater flexibility and control over multiple parameters.

## Conclusion

The performed studies demonstrate that the IMNGS platform is an innovative layer over the SRA repository for generation of novel insight in microbiology and microbiome research. Sample-specific integration and processing of all 16S rRNA gene amplicon studies available in sequence repositories offer a transparent, flexible, and scalable platform. In combination with a user friendly and intuitive interface, IMNGS represents a unique resource for ecological studies and the retrieval of selected microbial prevalence patterns and sequence-based diversity. Given the ever increasing number and volume of new amplicon sequencing projects, IMNGS will rapidly expand the coverage in range and depth of biomes profiles, allowing even more significant results in the near future.

## Methods

### IMNGS architecture and specifications

**Figure 3:**
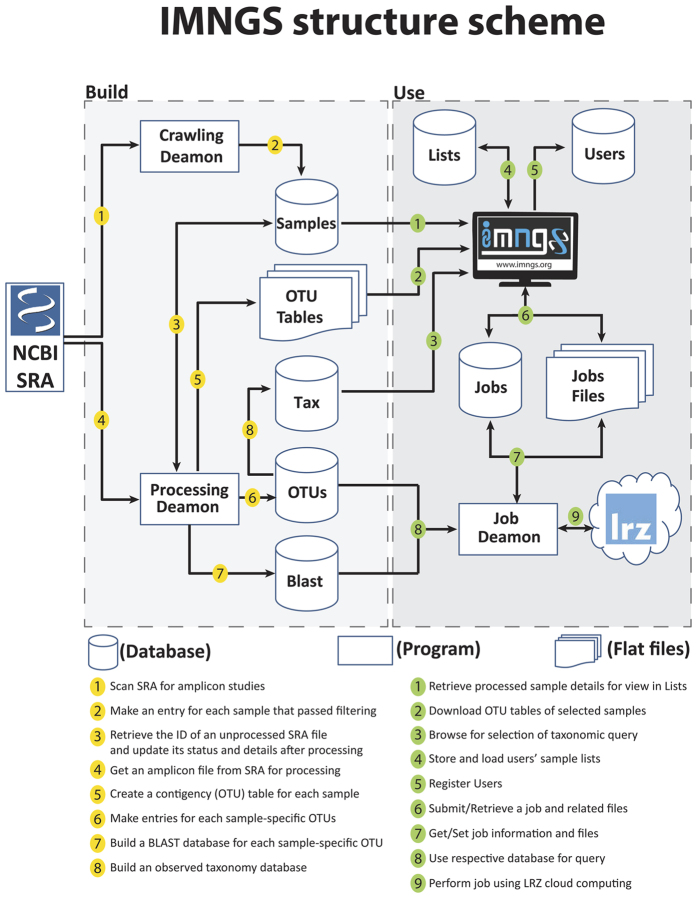
Overview of the IMNGS system. IMNGS can be separated into two components: the **Build**, where SRA files are retrieved and processed, and the **Use**, where users query and interact with data via the web front. The pipeline is fully automated and can run unsupervised, ensuring smooth integration of new data.

IMNGS is built as a freely available web platform. The web interface allows registered users to submit and manage jobs or directly download preprocessed sample-specific OTU tables and sequences. This is achieved through linkage to several local SQL databases and backbone operations written in Perl and Python. A comprehensive scheme of the IMNGS structure is available in Fig. 3. Registration to use the service is required in order to maintain data and personalized settings (*e.g.*, email notifications, manually selected sample lists). Due to the computation cost associated with operations that can be run with IMNGS, each user can have only one active job at a time. In case of multiple user requests, additional cloud computing nodes are recruited to serve the demand. Each job is allocated specific resources making parallel processing possible without substantial extension of running times. Nevertheless, in case the number of individual requests exceeds the system capacity (currently 4 local and 6 cloud nodes for a total of 10 parallel jobs processing), jobs are queued based on submission time in a first-come first-served fashion. Because sequence similarity searches are most time-consuming, especially when performed against the complete list of samples available in IMNGS, and in order to improve responsiveness of the system and avoid abuse, the maximum number of sequence per query was set to 10. Results are kept in the system for two weeks before automatic removal; therefore, prompt download of results upon completion is advised.

### Automated retrieval of 16S rRNA gene amplicon data from SRA

The ftp site of SRA is checked automatically on a weekly basis for samples that correspond to 16S rRNA gene amplicon studies. Selection is based on the attributes of library preparation such as the strategy (amplicon or other), source (metagenomic or other) and selection (PCR or other). In addition, a size filter is applied excluding studies with mean sequence length <200 nucleotides for single reads or <120 nucleotides for paired-end reads (counting only the relevant part of the read, *i.e.*, excluding primers, barcodes, etc.). Selected files are downloaded and an SQL record is created, including accessions and available sample information such as NCBI biosample taxonomy (origin of samples), sequence number per sample, or mean read length.

### Processing and classification of SRA datasets

Every SRA dataset (sample) that passes the initial filters is treated independently. Although SRA and ENA require studies to be submitted as independent demultiplexed samples, some cases of submission do not follow this rule. Since we have no means to automatically separate those cases, we process all datasets as individual samples as follows. First the samples are pre-filtered to remove non-16S rRNA amplicon datasets. For this purpose, the first 100 quality reads from each dataset are extracted and tested using sortmeRNA^35^ (version 2.0). If >10% of the reads are classified as 16S rRNA gene amplicons, the corresponding sample is kept for analysis or else discarded. All biological reads in SRA files longer than 200, or 120 nucleotides if paired, are exported as fastq files, using fastq-dump (SRA tools), and then analyzed with a modified version of the UPARSE pipeline^15^. This length cutoff was imposed to restrict the analysis to samples containing amplicons with sufficient resolution for phylogenetic studies and a relatively secure classification. This decision excludes by default all early days Illumina studies which delivered very short read lengths and/or non-overlapping reads, but keeps the rapidly outnumbering studies performed using Illumina MiSeq platforms. In more details, the exact pipeline is as follows: If reads originate from paired-end sequencing, they are truncated to the most upstream nucleotide position with quality ≤3 and merged. If the final read count per sample after assembly (and corresponding sequence filtering based on expected errors) is <1000 and forward reads were >200 nucleotides, samples are reprocessed as single reads. Single reads are trimmed to the mean read length in the given sample minus one standard deviation; original reads below this threshold length are discarded. Trimmed single or merged paired reads that have an expected error rate >2% are also discarded. Finally, if the final number of high quality reads after all filtering steps is <1000, the corresponding sample is excluded. The quality-filtered fastq reads are then dereplicated, sorted by abundance, and *de novo* clustered using USEARCH^36^ (32-bit version 8) at 97% sequence identity. The generated OTUs are checked for the presence of chimeras against the Ribosomal Database Project (RDP) database^37^ using UCHIME^38^. Non-prokaryotic OTUs (*e.g.*, phiX or eukaryotic contaminations) are filtered out using SortmeRNA^35^ (version 2.0), and the final OTU list is used to map filtered reads and build an OTU table with relative sequence abundance for each sample. The representative sequence of each OTU is used for taxonomically classification based on the RDP classifier^39^ (version 2.11 training set 15). Taxonomic calls with confidence >80% are added as extra taxonomy column in the OTU tables. Finally, all the collected information (OTU tables, sequences with corresponding taxonomic classification, origin, length) is parsed to the SQL databases, and UBLAST^36^ databases are created for future queries. Every single processed sample can be downloaded for inspection or comparison from the IMNGS site.

### Selection of personalized lists

An entry for each sample analyzed in IMNGS and its associated information (*e.g.* sample classification) is stored in an SQL database that can be viewed in the Lists tab of the web interface. The system offers several options for filtering and selecting lists of compatible samples that may be most appropriate for the type of analysis required. Hence, creation of lists assists in the biological interpretation of results by giving the chance to work on well-curated samples if desired, and also allows minimizing computation time. As an example, in order to create a list of all animal gut samples, we filtered all available datasets containing the word “gut” in their “Origin” and exported the list using the “Export” button. The exported spreadsheet file was then manually curated by removal of all samples with ambiguous host information. The remaining list of selected samples was imported and stored back to IMNGS as “Gut samples” list using the “Import” button. As IMNGS is continuously expanding, any lists get eventually outdated if not taken care of. If users create a special manually curated list of samples sharing defined characteristics and publish work created by using this list, we strongly recommend adding the list of samples as supplementary materials to the manuscript so that others can reuse it to reproduce data or address novel questions.

### Similarity query and prevalence analysis

To initiate a similarity query, a target sequence of interest is needed as template. Full or nearly-full length 16S rRNA gene sequence is recommended to ensure good coverage across all studies independent of 16S rRNA gene regions. For the purpose of demonstration, we used the 16S rRNA gene sequence of *Acetatifactor muris* (GenBank accession HM989805). In the Query/Similarity tab in IMNGS, we entered the FASTA formatted sequence of *A. muris*, selected the “Gut samples” as target list, and 95% as the sequence similarity threshold for query. Similarity queries are performed by IMNGS with UBLAST^36^ over the preformatted databases of sequences. Depending on the number of selected samples to be searched (all or lists), a similarity query can take up to 6 hours until completion. Results were downloaded from the Jobs tab as a compressed folder containing all output and information files (see Supplementary File S1 for detailed description of output files). Since the purpose of this demo search was to determine the host range of colonization without consideration of relative sequence abundances, the “report.0.tab” file was used, *i.e.* the report of positive samples per sample category (e.g. marine water, human skin, etc.) where a single hit is sufficient for a sample to be considered as positive. Different levels of relative abundances are offered to users, excluding rare (>0.1% threshold) or including only abundant (>1%) sequences. Detailed information on individual hits can be extracted from the “*.hits.tab” file, containing for instance sample type and matched sequence numbers for each query, and all hit sequences are available in “*.seqs.fasta” (the asterisk indicates the numbering of the sequence used as query).

### Taxonomic query and diversity analysis

To perform a taxonomic query, users must select the Query/Taxonomy tab and use the provided taxonomy tables to specify the target taxonomic group of interest. Available options cover all the RDP detected taxonomies of all processed sequences up to the current build. Users should always keep in mind that taxonomic classification among different systems (e.g. SILVA or Greengenes) or even among different builds of e.g. the RDP classifier may differ. For this reason, it is worth checking how a sequence belonging to the taxonomic group of interest would be classified by the RDP classifier release 2.11 (for the current version of IMNGS) before performing taxonomic queries. Results are retrieved from the SQL database, include all sequences assigned to the selected taxonomic rank and their abundance in each sample or sample categories, and can be downloaded from the Jobs tab. For demonstration, we used the bacterial phylum *Poribacteria* to determine its sequence-supported diversity and environmental profile. For completeness, we also extracted all 16S rRNA gene sequences classified as *Poribacteria* in GenBank. The complete set of sequences was aligned with SINA^40^ in SILVA^41^. Since not all studies available in SRA focus on the same 16S rRNA gene region, reads from different studies will often not overlap. Because diversity analysis requires homologue positions, the alignment region with highest coverage (number of non-gap bases) was selected and the rest of the alignment was trimmed. Sequences were first clustered into “species” groups (~97% similarity) using CROP^42^ (version 1.33), an unsupervised Bayesian clustering method with a soft cutoff boundary showing more conservative estimations of OTUs compared with other clustering methods like complete-linkage. The resulting centroid representatives were clustered at the “genus” level (~95% similarity) and then again at the “family” level (~90%). This created a hierarchical organization in a tree like structure, which was visualized using GraPhlAn^43^ (version 0.9.7). For the sake of comparison, sequences were clustered also with a complete-linkage method implemented in ESPRIT^44^ (version 1.4) using default settings. Details about the occurrence of each OTU and the contribution in each sample were extracted from the “taxonomic_occurrences.tab” using custom Perl scripts. For a more detailed description of taxonomic query output files, please refer to Supplementary File S2.

### Analysis of novel 16S rRNA gene amplicon datasets

Users’ raw amplicon datasets can be analyzed using the same pipeline used for processing SRA samples with some additional features, as detailed in own previously published work^45^,^46^. The pipeline is currently optimized for processing standard Illumina sequencing -derived fastq files. Denovo analysis allows demultiplexing the reads to their corresponding samples based on mapping files containing barcodes information. The implementation is fast, taking less than 1 hour per study (containing at least up to 200 samples). As in the case of SRA files, the similarity cutoff for clustering is set to 97% identity. Users have the freedom to select analysis parameters, including trimming options at both sequence ends (as they have been shown to contain biases), and filtering of OTU tables based on relative abundance thresholds (to exclude spurious OTUs that often appear in deep sequencing datasets). We recommend using sequencing controls (e.g. reagents only, mock communities, samples from germfree animals) in every project to assist in the selection of meaningful cutoffs for your experiment. For a detailed overview of analysis output files, please refer to Supplementary File S3. Instructions on input files (e.g. sequence orientation of barcodes) are provided online (www.imngs.org). If the Analysis feature of IMNGS is used for de novo processing of sequencing files that are not for comparison with preprocessed SRA samples, we recommend to reclassify the OTU sequences with the latest online RDP classifier and even improve taxonomic assignments by combining results generated by other taxonomic classification systems like SILVA^41^ and Greengenes^47^.

## Additional Information

**How to cite this article**: Lagkouvardos, I. *et al*. IMNGS: A comprehensive open resource of processed 16S rRNA microbial profiles for ecology and diversity studies. *Sci. Rep.* **6**, 33721; doi: 10.1038/srep33721 (2016).

## Supplementary Material

Supplementary File S1

Supplementary File S2

Supplementary File S3
