# Supplementary material for: IMNGS: A comprehensive open resource of processed 16S rRNA microbial profiles for ecology and diversity studies
Source: Sci Rep. 2016 Sep 23;6:33721. doi: 10.1038/srep33721 (PMC5034312; doi:10.1038/srep33721)
Supplement: Supplementary File S1 [file srep33721-s1.pdf]

Explanations for Similarity Query output files

Upload.txt

Recapitulates the sequences used for query by users. In the example below, sequences are truncated for space economy. For ecological studies, it is important to use 16S rRNA gene sequences as complete as possible to cover all possible variable regions contained in different SRA-derived sequencing projects.

Example

```
>HE978271
ATGCAAGTCGAGCGGATGAAGGGAGCTTGCTCCTGGATTCAGCGGCGGACGGGTGAGTAATGCCTAGGAATCTGCCTGGTAG ...
>U96927
ATCCAAGTCGAACGGCAGCACGGGTGCTTGCACCTGGTGGCGAGTGGCGAACGGGTGAGTAATACATCGGAACATGTCCTGT ...
>X71837
ATGCAAGTCGAACGAGGGCATTCTTTCGGGGATGTTCCTAGTGGCGGACGGGTGAGTAACGCGTGGGAATCTGCCTGATAGT ...
>Z29619
ATGCAAGTCGAACGAGGGTTTCCTTCGGGGGGCCTTAGTGGCGCACGGGTGAGTAACACGTGGGAACCTGCCTTTCGGTTC ...
>Z32635
ATGCAAGTCGAACGGCAGCATACTAGCTTGCTAGGTTGATGGCGAGTGGCGAACGGGTGAGTAACGCGTAGGAATATGCCTT ...
```

Seq_id_map.tab

The reference number assigned to each sequence in the query fasta file. All sequence files and results follow this conversion.

Example

```
1   HE978271
2   U96927
3   X71837
4   Z29619
5   Z32635
```

Counts_overview.tab

The number of hits (sequences) per sample and per query over all selected samples at the different similarity thresholds.

Example

```
#SampleID   Size    Description 1.99    2.99    1.97    2.97
SRR536792   476648  human gut metagenome    2452    0   2553    0
SRR389090   359248  human gut metagenome    340 0   342 6
SRR389091   343996  human gut metagenome    61  0   62  19
SRR514790   162501  human gut metagenome    6   0   6   0
SRR505779   3139    human gut metagenome    5   0   5   0
SRR639158   22880   human gut metagenome    3   0   3   0
```

This example shows the number of query-like sequences in several samples. Size indicates the number of total sequences in the corresponding SRA sample (can be used to calculate relative abundances). Description refers to the taxonomy of the sample in SRA.
It can be seen that sequence 1 (1.99, 1.97) can be found in the selected samples, but not sequence 2 (2.99, 2.97). Moreover, sequences corresponding to query 1 are almost exclusively matches at the strain level, as the number of hits did not increase after relaxing the similarity threshold from 99% to 97% (1.99 vs. 1.97).

Selected_db_list.txt

The list of samples that were set for query. The easiest way for third parties to repeat the query is to send this list and then import it in IMNGS.

Example

```
ERR174134
ERR174136
ERR174137
ERR174138
ERR174139
ERR174140
ERR174141
```

#.hits.tab

This file contains the UBLAST output of the hits for each query in tabular format (e.g., 1.hits.fasta). It can be used to trace back OTUs matching the query in all SRA samples analyzed.

Example

```
Query_name  Target_seq  Identity    Al_length   mismatches  gaps    Query_start Query_end   Target_start    Target_end  E-value Bi
HE978271    SRR514792.174404.2;size=1;tax=...   98.5    194 3   0   281 474 21  214 1.1e-94 342.8
HE978271    SRR389090.3588.3;size=340;tax=...   100.0   435 0   0   39  473 435 1   2.4e-233    804.4
HE978271    SRR389090.290961.3;size=2;tax=...   97.5    401 5   5   75  473 398 1   1.6e-175    612.4
HE978271    SRR578501.5520.4;size=1;tax=... 100.0   326 0   0   523 848 326 1   9.3e-175    603.1
HE978271    SRR578403.2695.4;size=1;tax=... 97.5    244 6   0   605 848 244 1   8.1e-120    418.5
HE978271    SRR639307.16357.2;size=3;tax=...    98.8    329 3   1   41  369 6   333 4.9e-164    571.7
HE978271    SRR389091.8838.3;size=61;tax=...    100.0   433 0   0   41  473 433 1   3.3e-232    800.7
```

#.seqs.fasta

This file contains all fasta sequences of the hits for each query (e.g., 1.seqs.fasta). The sequence description line is semicolon delimited (;) and contains the ID of the centroid sequence representing the OTU cluster, the number of sequences that clustered under that OTU, and the taxonomy assigned to the OTU by RDP classifier.

Example

```
>SRR360670.857.2;size=1;tax=Bacteria;Proteobacteria;Betaproteobacteria;Burkholderiales;Burkholderiaceae;Burkholderia;
TCTCCATCCATCAGAGTTTGATCATGGCTCAGATTGAACGCTGGCGGCATGCCTTACACATGCAAGTCGAACGGCAGCAC ...
>SRR360615.436.2;size=1;tax=Bacteria;Proteobacteria;Betaproteobacteria;Burkholderiales;Burkholderiaceae;Burkholderia;
TCTAATGCGCTCAGAGTTTGATCCTGGCTCAGATTGAACGCTGGCGGCATGCCTTACACATGCAAGTCGAACGGCAGCAC ...
>SRR360638.1757.2;size=1;tax=Bacteria;Proteobacteria;Betaproteobacteria;Burkholderiales;Burkholderiaceae;Burkholderia;
TCTTGGTTCGTCAGAGTTTGATCCTGGCTCAGATTGAACGCTGGCGGCATGCCTTACACATGCAAGTCGAACGGCAGCAC ...
>SRR360619.1405.2;size=3;tax=Bacteria;Proteobacteria;Betaproteobacteria;Burkholderiales;Burkholderiaceae;Burkholderia;
TCGTTCCTTGTCAGAGTTTGATCATGGCTCAGATCGAACGCTGGCGGCATGCCTTACACATGCAAGTCGAACGGCAGCAC ...
```

Report.#.tab

The number of samples that were positive for the presence of query-like sequences for each sample category.

1. **Report.0.tab**
   An SRA-derived sample is considered positive if the query-like sequences sum up to more than 0% of the total number of sequences in that sample (i.e. any abundance).

2. **Report.0.1.tab**
   An SRA-derived sample is considered positive if the query-like sequences sum up to more than 0.1% of the total number of sequences in that sample (i.e. excluding rare abundances).

3. **Report.1.tab**
   An SRA-derived sample is considered positive if the query-like sequences sum up to more than 1% of the total number of sequences in that sample (i.e. including only dominant OTUs).

Example

```
Environment_source	Samples_number	1.99	2.99	1.97	2.97
human gut metagenome	833	9	0	14	0
soil metagenome	135	0	19	0	48
```

In this example, 968 samples were queried covering two environmental types (human gut and soil) as shown in the first column. The second column shows the exact number of samples in each category followed by the number of samples positive for the presence of query-like sequences. This example is supportive of bacterium 1 and 2 to be specific for human gut and soil, respectively.
