# Supplementary material for: IMNGS: A comprehensive open resource of processed 16S rRNA microbial profiles for ecology and diversity studies
Source: Sci Rep. 2016 Sep 23;6:33721. doi: 10.1038/srep33721 (PMC5034312; doi:10.1038/srep33721)
Supplement: Supplementary File S2 [file srep33721-s2.pdf]

Explanations for Taxonomic Query output files

Selected_dbs_list.txt

The list of samples that were selected for this taxonomic query. The easiest way for third parties to repeat the query is to share lists so they can be imported in IMNGS by others.

Example

```
ERR174134
ERR174136
ERR174137
ERR174138
ERR174139
ERR174140
ERR174141
```

taxonomic_occurancies.tab

A tab delimited file containing the following information:

1) The sample name
2) The sample category as deposited in SRA
3) The total number of quality sequences in the sample after filtering
4) The number of OTUs that matched the target taxonomy
5) The number of sequences from this sample that clustered under the positive OTUs

Example

```
Sample(run) Sample_category Sample_seq_num  Target_Tax_OTUs Target_Tax_seqs
ERR185980   human gut metagenome    24363   47  102
ERR185982   human gut metagenome    4116    0   0
ERR185991   human gut metagenome    2427    0   0
SRR360592   human gut metagenome    707 0   0
```

seqs_matching_taxonomy.fasta

This file contains all the fasta sequences of the OTUs matching the selected taxonomy. The sequence description line is semicolon delimited (;) and contains the ID of the centroid sequence representing the OTU cluster, the number of sequences that clustered under that OTU, and the taxonomy assigned to the OTU by RDP classifier.

Example

```
>SRR360670.857.2;size=1;tax=Bacteria;Proteobacteria;Betaproteobacteria;Burkholderiales;Burkholderiaceae;Burkholderia;
TCTCCATCCATCAGAGTTTGATCATGGCTCAGATTGAACGCTGGCGGCATGCCTTACACATGCAAGTCGAACGGCAGCAC ...
>SRR360615.436.2;size=1;tax=Bacteria;Proteobacteria;Betaproteobacteria;Burkholderiales;Burkholderiaceae;Burkholderia;
TCTAATGCGCTCAGAGTTTGATCCTGGCTCAGATTGAACGCTGGCGGCATGCCTTACACATGCAAGTCGAACGGCAGCAC ...
>SRR360638.1757.2;size=1;tax=Bacteria;Proteobacteria;Betaproteobacteria;Burkholderiales;Burkholderiaceae;Burkholderia;
TCTTGGTTCGTCAGAGTTTGATCCTGGCTCAGATTGAACGCTGGCGGCATGCCTTACACATGCAAGTCGAACGGCAGCAC ...
>SRR360619.1405.2;size=3;tax=Bacteria;Proteobacteria;Betaproteobacteria;Burkholderiales;Burkholderiaceae;Burkholderia;
TCGTTCCTTGTCAGAGTTTGATCATGGCTCAGATCGAACGCTGGCGGCATGCCTTACACATGCAAGTCGAACGGCAGCAC ...
```

Report.#.tab

A tab delimited file with the number of samples that were positive for the presence of the queried taxonomy for each sample category.

1. **Report.0.tab**
   A sample is considered positive if sequences classified in the target taxonomy sum up to more than 0% of the total number of sequences in that sample (any abundance).

2. **Report.0.1.tab**
   A sample is considered positive if sequences classified in the target taxonomy sum up to more than 0.1% of the total number of sequences in that sample (i.e. excluding rare abundances).

3. **Report.1.tab**
   A sample is considered positive if sequences classified in the target taxonomy sum up to more than 1% of the total number of sequences in that sample (i.e. including only dominant OTUs).

Example

```
Environment_source  Number_of_samples   Taxonomy_positive
human gut metagenome    833 159
soil metagenome 135 0
```

In this example, 968 samples were queried covering two environmental types (human gut and soil) as shown in the first column. The second column shows the exact number of samples in each category, followed by the number of samples positive for the presence of sequences classified as the targeted taxonomy (third column).
