# Supplementary material for: IMNGS: A comprehensive open resource of processed 16S rRNA microbial profiles for ecology and diversity studies
Source: Sci Rep. 2016 Sep 23;6:33721. doi: 10.1038/srep33721 (PMC5034312; doi:10.1038/srep33721)
Supplement: Supplementary File S3 [file srep33721-s3.pdf]

Explanations for Analysis output files

Analysis_Description.txt

This file contains a description of the settings and the methods used for analysis of the raw reads into clusters of OTUs. It contains three sections: input; settings; methods.

Example

```
Log of the AnalysisName study analysis.
Analysis Description: This is an analysis conducted as an example

Input
=======================================================================
Run Name:Standard.
Are the reads paired: Yes.
The run has two indexes: Yes.
The reverse index filename (I1) is: I1.fastq.
The forward index filename (I2) is: I2.fastq.
The forward reads filename (R1) is: R1.fastq.
The reverse reads filename (R2) is: R2.fastq.

Settings
=======================================================================
Number of allowed mismatches in the barcode: 2
Min fastq quality score for trimming of unpaired reads: 3
Min length of the paired sequence cutoff: 400
Max length of the paired sequence cutoff: 500
Max number of expected errors in paired sequences: 3
Length of trimming at the forward side of the seqs: 10
Length of trimming at the reverse side of the seqs: 10
Min relative abundance of OTU cutoff (0-1): 0.0025

Methods
=======================================================================
This is a UPARSE based analysis pipeline (PMID:23955772).
Demultiplexing was performed with demultiplexor_v4.pl (Unpublished Perl script).
Pairing, quality filtering and OTU clustering was done using USEARCH7 (PMID:20709691).
Chimeras were filtered by using UCHIME (PMID:21700674) (with RDP set 10 as a reference database).
Taxonomic classification was based on RDP classifier version 2.10 training set 10 (PMID:17586664).
Sequence were aligned with MUSCLE (PMID:15034147) and the tree constructed using Fasttree(PMID:20224823).

### If you publish these results, please cite the above software. ###
=======================================================================
```

OTUs-Seqs.fasta

This file contains all the sequences of the cluster centroids (OTUs)

Example

```
>OTU_1
GCTGCAGTGGGGAATATTGCACAATGGGGGAACCCTGATGCAGCGACGCCGCGTGAGTG...
>OTU_2
GCTGCAGTGGGGAATATTGCACAATGGGGAAACCCTGATGCAGCGACGCCGCGTGAGCG...
>OTU_3
GCTGCAGTGGGGAATATTGCACAATGGGGAAACCCTGATGCAGCGACGCCGCGTGAAGG...
```

OTUs-Table.tab

A tab delimited file with the number of reads per sample that clustered within each OTU. All OTUs were taxonomically classified using the RDP classifier and the result was added in a taxonomy column at the right hand side of the table.

Example

```
#OTUId  Sample1 Sample2 taxonomy
OTU_4   11894   7648    Bacteria;Firmicutes;Bacilli;Lactobacillales;Enterococcaceae;Enterococcus;
OTU_9   10399   7381    Bacteria;Firmicutes;Bacilli;Bacillales;Bacillaceae 1;Bacillus;
OTU_5   9710    7256    Bacteria;Firmicutes;Erysipelotrichia;Erysipelotrichales;Erysipelotrichaceae;Clostridium XVIII;
OTU_16  9683    7626    Bacteria;Firmicutes;Bacilli;Bacillales;Staphylococcaceae;Staphylococcus;
OTU_1   7991    8976    Bacteria;Firmicutes;Clostridia;Clostridiales;Lachnospiraceae;;
OTU_6   6871    7912    Bacteria;Firmicutes;Clostridia;Clostridiales;Ruminococcaceae;Flavonifractor;
OTU_8   6020    8384    Bacteria;Actinobacteria;Actinobacteria;Coriobacteriales;Coriobacteriaceae;Enterorhabdus;
OTU_11  4771    7765    Bacteria;Bacteroidetes;Bacteroidia;Bacteroidales;Porphyromonadaceae;Parabacteroides;
OTU_20  4486    7858    Bacteria;Bacteroidetes;Bacteroidia;Bacteroidales;Bacteroidaceae;Bacteroides;
OTU_14  3505    6868    Bacteria;Proteobacteria;Gammaproteobacteria;Pseudomonadales;Pseudomonadaceae;Pseudomonas;
OTU_10  3407    7501    Bacteria;Bacteroidetes;Bacteroidia;Bacteroidales;Rikenellaceae;Alistipes;
OTU_15  2733    6906    Bacteria;Actinobacteria;Actinobacteria;Actinomycetales;Actinomycetaceae;Actinomyces;
OTU_284 288 577 Bacteria;Bacteroidetes;Bacteroidia;Bacteroidales;Bacteroidaceae;Bacteroides;
OTU_19  148 6450    Bacteria;Actinobacteria;Actinobacteria;Actinomycetales;Promicromonosporaceae;Cellulosimicrobium;
OTU_18  19  13  Bacteria;Bacteroidetes;Bacteroidia;Bacteroidales;Bacteroidaceae;Bacteroides;
OTU_2   18  21  Bacteria;Firmicutes;Clostridia;Clostridiales;Lachnospiraceae;Roseburia;
OTU_12  12  12  Bacteria;Firmicutes;Clostridia;Clostridiales;Ruminococcaceae;Faecalibacterium;
OTU_22  8   13  Bacteria;Bacteroidetes;Bacteroidia;Bacteroidales;Bacteroidaceae;Bacteroides;
OTU_7   8   6   Bacteria;Firmicutes;Clostridia;Clostridiales;Lachnospiraceae;;
OTU_21  6   13  Bacteria;Firmicutes;Clostridia;Clostridiales;Lachnospiraceae;Clostridium XlVa;
OTU_17  6   0   Bacteria;Firmicutes;Clostridia;Clostridiales;Lachnospiraceae;Lachnospiracea_incertae_sedis;
```

OTUs-Tree.tre

This is a rooted tree (Newick format) calculated with fasttree (Maximum Likelihood approximation). A true ML tree can be produced with other common phylogenetic suits using the OTU sequences provided.

Barcodes mapping file

This file (with the name of the corresponding analysis) contains the barcodes for demultiplexing of the raw reads and generation of the OTU table.
A detailed mapping file template with explanations is available on the webfront.

Example

```
#Sample BarcodeSequence.reverse BarcodeSequence.forward
Sample1 CTCGACTT    AAGCAGCA
Sample2 CTCGACTT    ACGCGTGA
```

This example corresponds to a doubled-index paired-end sequencing project and thus contains two columns for barcode sequences.

stats.tab

A file recapitulating the number of sequences that pass each step of the pipeline for each sample.

Example

```
Sample1 106984
Sample2 125327
```
